# When the Brain Takes ‘BOLD’ Steps: Real-Time fMRI Neurofeedback Can Further Enhance the Ability to Gradually Self-regulate Regional Brain Activation

**DOI:** 10.1016/j.neuroscience.2016.09.026

**Published:** 2018-05-15

**Authors:** Bettina Sorger, Tabea Kamp, Nikolaus Weiskopf, Judith Caroline Peters, Rainer Goebel

**Affiliations:** a Department of Cognitive Neuroscience, Maastricht University, Maastricht, The Netherlands; b Maastricht Brain Imaging Center (M-BIC), Maastricht, The Netherlands; c Department of Neurophysics, Max Planck Institute for Human Cognitive and Brain Sciences, Leipzig, Germany; d Wellcome Trust Centre for Neuroimaging, UCL Institute of Neurology, University College London, London, United Kingdom; e Department of Neuroimaging and Neuromodeling, Netherlands Institute for Neuroscience, An institute of the Royal Netherlands Academy of Arts and Sciences (KNAW), Amsterdam, The Netherlands

**Keywords:** BOLD, blood oxygenation level-dependent, EPI, echo-planar imaging, (f)MRI, (functional) magnetic resonance imaging, (f)NIRS, (functional) near-infrared spectroscopy, LIS, ‘locked-in’ syndrome, ROI(s), region(s) of interest, rtfMRI, real-time functional magnetic resonance imaging, VOI(s), volume(s) of interest, (gradual) self-regulation, mental tasks, cognitive strategies, (real-time) functional magnetic resonance imaging, neurofeedback, brain-computer interface

## Abstract

Brain-computer interfaces (BCIs) based on real-time functional magnetic resonance imaging (rtfMRI) are currently explored in the context of developing alternative (motor-independent) communication and control means for the severely disabled. In such BCI systems, the user encodes a particular intention (*e.g.*, an answer to a question or an intended action) by evoking specific mental activity resulting in a distinct brain state that can be decoded from fMRI activation. One goal in this context is to increase the degrees of freedom in encoding different intentions, *i.e.*, to allow the BCI user to choose from as many options as possible. Recently, the ability to voluntarily modulate spatial and/or temporal blood oxygenation level-dependent (BOLD)-signal features has been explored implementing different mental tasks and/or different encoding time intervals, respectively. Our two-session fMRI feasibility study systematically investigated for the first time the possibility of using magnitudinal BOLD-signal features for intention encoding. Particularly, in our novel paradigm, participants (*n* = 10) were asked to alternately self-regulate their regional brain-activation level to 30%, 60% or 90% of their maximal capacity by applying a selected activation strategy (*i.e.*, performing a mental task, *e.g.*, inner speech) and modulation strategies (*e.g.*, using different speech rates) suggested by the experimenters. In a second step, we tested the hypothesis that the additional availability of feedback information on the current BOLD-signal level within a region of interest improves the gradual-self regulation performance. Therefore, participants were provided with neurofeedback in one of the two fMRI sessions. Our results show that the majority of the participants were able to gradually self-regulate regional brain activation to at least two different target levels even in the absence of neurofeedback. When provided with continuous feedback on their current BOLD-signal level, most participants further enhanced their gradual self-regulation ability. Our findings were observed across a wide variety of mental tasks and across clinical MR field strengths (*i.e.*, at 1.5 T and 3 T), indicating that these findings are robust and can be generalized across mental tasks and scanner types. The suggested novel parametric activation paradigm enriches the spectrum of current rtfMRI-neurofeedback and BCI methodology and has considerable potential for fundamental and clinical neuroscience applications.

## Highlights

• Humans are able to gradually self-regulate regional brain activation by applying cognitive strategies.
• Providing rtfMRI neurofeedback can enhance the gradual self-regulation ability.
• Findings are generalizable to various mental tasks and clinical MR field strengths.
• Novel parametric activation paradigm enriches spectrum of rtfMRI-neurofeedback and BCI methodology.

## Introduction

Real-time functional magnetic resonance imaging (rtfMRI) allows for brain-computer interfacing – therewith, providing a tool to monitor and alter current a) *brain activation* (both regionally [*e.g.*, Caria et al., 2007, Zotev et al., 2011, Linden et al., 2012, Canterberry et al., 2013, Greer et al., 2014, Young et al., 2014b, Cordes et al., 2015] and in widely distributed regions [*e.g.*, LaConte et al., 2007]) or b) *brain connectivity patterns* (*e.g.*, Koush et al., 2013, Kim et al., 2015). RtfMRI research focuses on two application possibilities of brain-computer interfaces (BCIs): neurofeedback learning/therapy and brain-based communication and control (Goebel et al., 2010).

Since the introduction of the rtfMRI method in 1995 (Cox et al., 1995), numerous studies have investigated its suitability for neurofeedback applications. In several proof-of-principle studies with healthy participants, it has been shown that different kinds of overt (behavioral) and covert (mental) tasks can be used to voluntarily in- or decrease (up- or downregulate) the blood oxygenation level-dependent (BOLD) signal in various cortical brain regions, including sensory (*e.g.*, Haller et al., 2010, Scharnowski et al., 2012, Robineau et al., 2014, Auer et al., 2015), (pre)motor (*e.g.,* Yoo and Jolesz, 2002, deCharms et al., 2004, Berman et al., 2012, Chiew et al., 2012), insular (*e.g.,* Caria et al., 2007, Berman et al., 2013, Ruiz et al., 2013b, Emmert et al., 2014), anterior cingulate (*e.g.,* deCharms et al., 2005, Canterberry et al., 2013, Emmert et al., 2014, Guan et al., 2015), posterior cingulate (Garrison et al., 2013a, Garrison et al., 2013b, Zhang et al., 2013b), dorsolateral prefrontal (Zhang et al., 2013a, Sherwood et al., 2016), inferior frontal (Rota et al., 2009) and orbitofrontal cortex (Hampson et al., 2012, Scheinost et al., 2013), as well as subcortical structures, including nucleus accumbens (Greer et al., 2014), amygdala (*e.g.,* Zotev et al., 2011, Brühl et al., 2014, Young et al., 2014b), striatum (Kirsch et al., 2015), substantia nigra (Sulzer et al., 2013b), and ventral tegmental area (MacInnes et al., 2016). As an extension, translational studies explored the feasibility of rtfMRI neurofeedback to remediate pathological brain activation associated with symptoms of various (mostly neurological and psychiatric) disorders including major depressive disorder (Linden et al., 2012, Young et al., 2014b, Hamilton et al., 2016, Zotev et al., 2016), schizophrenia (Ruiz et al., 2013a, Cordes et al., 2015), Parkinson’s disease (Subramanian et al., 2011), spider phobia (Zilverstand et al., 2015), chronic pain (deCharms et al., 2005, Guan et al., 2015), tinnitus (Haller et al., 2010), addiction (Canterberry et al., 2013, Li et al., 2013, Karch et al., 2015, Kirsch et al., 2015, Hartwell et al., 2016), obesity (Frank et al., 2012), autism (Caria and de Falco, 2015), and stroke (Chiew et al., 2012, Young et al., 2014a).

The second application possibility of rtfMRI, the employment of BCIs for motor-independent communication and control, also has considerable societal impact – being potentially of great importance for the severely disabled (*e.g.*, ‘locked-in’ syndrome [LIS] patients). For almost 30 years now, most BCI researchers have focused on developing communication and control BCIs based on *neuroelectric* signals (Farwell and Donchin, 1988, Chapin et al., 1999, Leuthardt et al., 2004, Scherer et al., 2004, Ramsey et al., 2006, Mellinger et al., 2007). Though these ‘classic’ BCIs (mostly based on electroencephalography [EEG]) have already been applied successfully in affected patients (Birbaumer et al., 1999, Kübler et al., 1999, Hochberg et al., 2006, Hochberg et al., 2012, Nijboer et al., 2008), not all individuals achieve proficiency in EEG-based BCI control (a phenomenon coined ‘BCI illiteracy’). Therefore, exploiting *hemodynamic* brain signals as measured with fMRI or functional near-infrared spectroscopy (fNIRS) has been suggested as an alternative approach (Weiskopf et al., 2003). One important aspect when developing communication and control BCIs is to try to increase the degrees of freedom in encoding different intentions, *i.e.*, to allow the BCI user to choose from as many options as possible. One necessity in this context is to enable the BCI user to voluntarily evoke just as many differentiable brain states (*e.g.*, distinct fMRI brain-activation patterns). But how can this be achieved?

Several approaches have been explored in the context of fMRI-based brain-computer interfacing: a first approach employed the modulation of *spatial* BOLD-signal features for encoding separate intentions by implementing different mental tasks (and thereby evoking spatially different brain-activation patterns). This possibility was tested in several fMRI experiments including proof-of-principle studies with healthy participants (Lee et al., 2009, Yoo et al., 2012) and clinical studies involving patients suffering from a disorder of consciousness in order to detect residual conscious awareness (Owen et al., 2006, Monti et al., 2010). In one study, healthy participants navigated through a two-dimensional (2D) virtual maze by performing a specific mental task (eliciting a unique brain-activation pattern) for each of the four movement directions (“right”, “left”, “up”, and “down”) (Yoo et al., 2004). In a later follow-up study, it was shown that this procedure also enables adequate control over 2D movements of a robotic arm (Lee et al., 2009). Note however, that the amount of mental tasks suited for encoding different intentions seems to be rather limited when using MRI scanners with conventional field strengths (1.5 T or 3 T). So far, the most successfully implemented mental tasks in this context are motor imagery, spatial navigation, mental calculation, and inner speech (Yoo et al., 2004, Owen et al., 2006, Boly et al., 2007, Lee et al., 2009). As a second approach to increase the degrees of freedom in encoding separate intentions, researchers have explored the possibility to systematically vary *temporal* BOLD-signal features (*i.e.*, using different encoding time intervals) (Sorger et al., 2009, Bardin et al., 2011). Finally, a combined use of both *spatial* and *temporal* BOLD-signal features was successfully tested and further developed to allow for encoding all letters of the English alphabet and the blank space enabling fMRI-based free-letter spelling (Sorger et al., 2012). Theoretically (and as a third option), it might be feasible to hemodynamically encode separate intentions by systematically varying the BOLD-signal level (*i.e.*, exploiting *magnitudinal* BOLD-signal features) within the same region of interest (ROI). The ability to differentially modulate the BOLD-signal level might be given *a priori* when instructing participants appropriately. However, providing neurofeedback on the current brain-activation level might further enhance the gradual self-regulation performance. Magnitudinal BOLD-signal features have been employed previously in a real-time ‘brain pong’ hyperscanning study (Goebel et al., 2004) where two interacting participants played pong by controlling the vertical position of their rackets by modulating the level of regional brain activation. In this game-like situation, gradual self-regulation of the BOLD signal was, however, not systematically investigated.

Based on the presented background, the current feasibility study investigated systematically whether healthy participants are able to gradually modulate the BOLD-signal level by employing different mental strategies and whether fMRI-based neurofeedback can facilitate the presumed gradual self-regulation ability (in the following coined *instantaneous feedback effect* to differentiate it from a*, e.g., feedback-transfer effect*).

In order to answer the abovementioned questions, participants were trained to modulate their BOLD-signal magnitude to different target levels without and with the support of rtfMRI neurofeedback about the BOLD-signal level in a predefined mental task-related brain region.

The main hypotheses of the current study were:

(1) The BOLD-signal level can be self-regulated gradually (*gradual self-regulation effect*).
(2) The availability of neurofeedback about the current BOLD-signal level further improves the gradual self-regulation performance (*instantaneous feedback effect*).

## Experimental procedures

### Participants

Ten healthy participants (age: 27 ± 3.8 years, five female, one left-handed), all students or staff members of the *Faculty of Psychology and Neuroscience* at *Maastricht University* with normal or corrected-to-normal vision participated in the study (see Table 1 for participants’ characteristics). None of the participants had participated in a neurofeedback experiment before. Before each MRI scanning session, participants gave written informed consent. The experimental procedure was approved by the local *Ethics Committee of the Faculty of Psychology and Neuroscience at Maastricht University*.

**Table 1:** Participants’ characteristics and methodological details

Participant	Sex	Age	Condition of 1st MRI session	MRI scanner (field strength)	Activation strategy (mental task)
P01^∗^	Male	24	Feedback	3T	Inner speech
P02	Male	27	Feedback	1.5T	Mental orchestra
P03	Female	32	No feedback	1.5T	Inner speech
P04	Female	35	No feedback	1.5T	Visual motion imagery
P05	Female	25	No feedback	1.5T	Inner speech
P06	Male	25	Feedback	3T	Mental drawing
P07	Male	28	Feedback	3T	Inner speech
P08	Male	23	Feedback	3T	Mental sounds
P09	Female	25	No feedback	1.5T	Mental running
P10	Female	26	No feedback	3T	Inner speech

*Remark:*^∗^left-handed.

### Experimental design

**Fig. 1:**
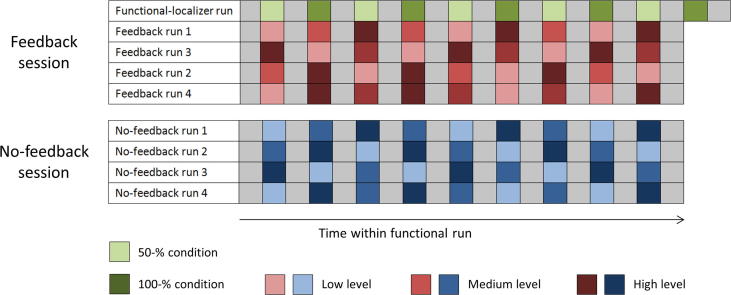
Overview of experimental design. The figure depicts the experimental design for one participant. Bluish and reddish colors indicate no-feedback and feedback conditions, respectively. Greenish colors refer to the two conditions implemented in the functional-localizer run. Resting blocks are indicated by gray cells. Resting and modulation blocks took 26 s each.

Participants were asked to modulate their BOLD signal to three different target levels. Importantly, participants received no feedback in one fMRI session, whereas in the other session they were provided with neurofeedback information on the current BOLD-signal level in a pre-defined mental task-related brain region. Thus, we employed a two-way within-subject design with *target level* (*low*, *medium* and *high*) and *type of training* (*no feedback* and *feedback*) as factors. For each participant, the no-feedback and feedback fMRI sessions were on separate days. Note that the order of the type-of-training conditions (no feedback-feedback or feedback-no-feedback) was balanced across participants (see Table 1) in order to exclude potential confounds. Both scanning sessions consisted of four training (modulation) runs (see Fig. 1) in which participants were visually instructed to modulate their BOLD-signal magnitude to the three different target levels. Each target-level condition appeared three times per run in randomized order resulting in a total of twelve trials per target-level and type-of-training condition. The duration of the nine modulation trials per run as well as of the intermingled ten resting periods was 26 s resulting in a modulation-run length of 8 min and 14 s. A feedback scanning session started with a functional-localizer run in order to select a mental task-specific neurofeedback target region. In the functional localizer, two target levels (*50%* and *100%*) were implemented (five trials per target-level condition). The two target-level conditions appeared in alternating order. Again, the duration of the (ten) modulation trials and the (11) resting periods were 26 s adding up to a total run duration of 9 min and 6 s.

### Visual instruction and neurofeedback presentation

**Fig. 2:**
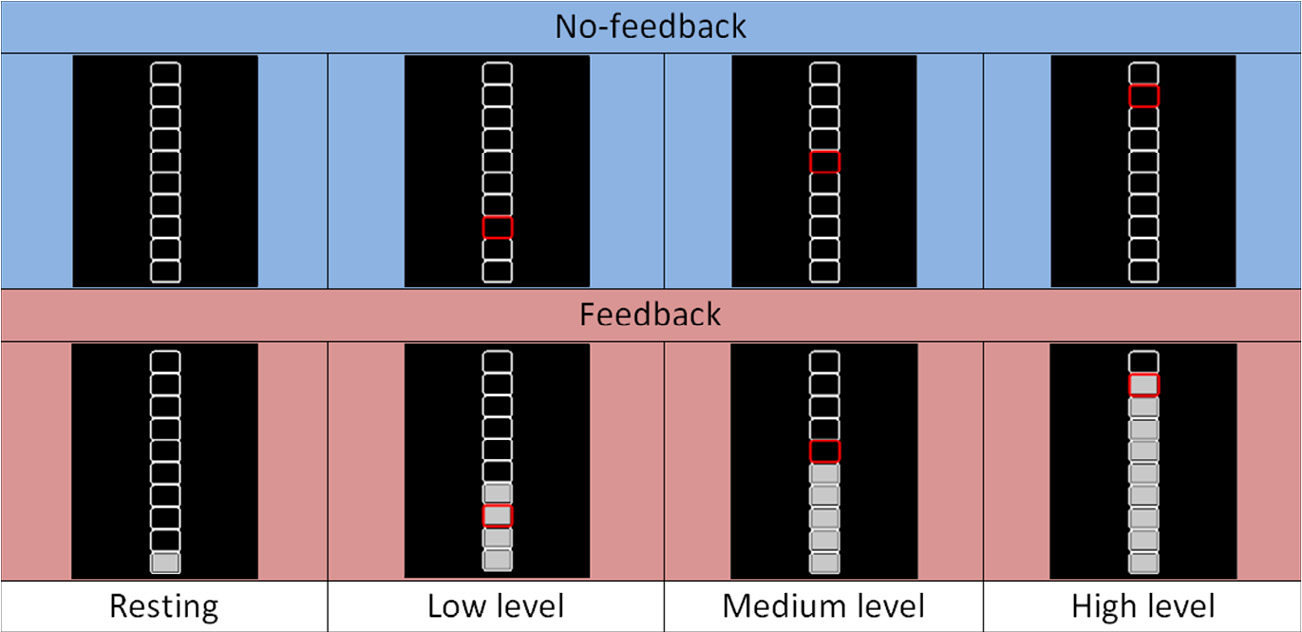
Visual instruction and neurofeedback display. A thermometer-like display on black background was used consisting of ten white rectangles stacked on top of each other. To instruct participants to adjust their BOLD signal to a particular target level, the outline of a certain rectangle turned red for the duration of the modulation trial. During resting blocks no rectangle was colored red. During feedback runs, continuously updated gradual feedback information was additionally provided by filling the rectangles with gray color according to the current BOLD signal intensity reached by the participant in the neurofeedback target region.

In order to instruct participants, a thermometer-like display on black background was used consisting of ten white rectangles stacked on top of each other (see Fig. 2). To instruct participants to adjust their BOLD signal to a particular target level, the outline of a certain rectangle turned red for the duration of the modulation trial. Thus, the vertical position of the colored rectangle represented the desired target level.

In the functional-localizer run, rectangle 5 (counted from bottom) corresponded to the 50-% condition and rectangle 10 represented the 100-% condition. In the modulation runs, rectangles 3, 6, and 9, corresponded to the low, medium and high target-level conditions, respectively. During resting periods, no rectangle was colored red.

In the modulation runs of the feedback session, participants were additionally provided with continuously updated gradual information about their current BOLD-signal level within the neurofeedback target region. This was realized by filling in (with gray color) the thermometer’s rectangles in such a way that the vertical position in the display corresponded to the *actual* BOLD-signal level within the neurofeedback target region. Note that the neurofeedback display was kept as intuitive as possible assuming that a straightforward interpretation of the neurofeedback information facilitates learning (Weiskopf et al., 2004b).

Visual stimulation was generated by a personal computer (PC) using custom-made software and projected onto a frosted screen located at the end of the scanner bore (at the side of the participant’s head) with a liquid crystal display (LCD) projector. Participants viewed the screen via a mirror mounted to the head coil at an angle of ∼45°.

### General procedure

#### Preparation

At the beginning of the first session, participants were familiarized with the general idea of the study (investigating the ability to reach different brain-activation levels without and with neurofeedback). They were introduced to the fMRI-neurofeedback concept and methodology and the general procedure of the current study. Furthermore, participants were familiarized with the neurofeedback display, hemodynamic delay and noise level of fMRI signals. Finally, they were instructed to avoid body movements while lying in the MRI scanner.

#### Suggestion and selection of activation and modulation strategies

Experimenters suggested various mental tasks (inner speech, motor imagery, mental calculation, visual imagery and auditory imagery) that had been proven to evoke robust brain activation in circumscribed brain regions in previous fMRI studies (*e.g.*, Yoo et al., 2004, Owen et al., 2006, Boly et al., 2007, Lee et al., 2009, Sorger et al., 2009, Sorger et al., 2012, Monti et al., 2010, Bardin et al., 2011) as possible *activation strategies*. Additionally, the experimenters recommended several *modulation strategies* that could be applied by participants to alter the brain-activation level. Basically, these strategies allowed for changing certain aspects of mental-task performance parametrically (*e.g.*, the speed, intensity or complexity). The modulation strategies were either based on neuroscientific pre-knowledge, *i.e.*, studies showing parametric effects on brain activation by systematically changing aspects of mental-task performance (*e.g.*, Culham and Kanwisher, 2001, Shergill et al., 2002, Berman et al., 2012, Lipp et al., 2012) or on naïve hypotheses of the experimenters on how the BOLD signal might be altered.

Participants were asked to choose an individual activation strategy which they could execute continuously and manipulate by applying the modulation strategies suggested by the experimenter (see above). Participants selected their activation strategies and *initial* modulation strategies based on personal preference or feeling of best mastery.

#### Task instruction

Participants were instructed to keep their selected activation strategy constant across all functional runs (functional-localizer, no-feedback and feedback runs). Thus, they should not change their general activation strategy across time (and sessions). In order to modulate their BOLD signal to the different target levels, participants were asked to apply the modulation strategies. Importantly, in the feedback condition participants were instructed to consider the provided neurofeedback information and to explore which of the modulation strategies were *most* effective. Moreover, participants were explicitly allowed to adapt the suggested modulation strategies or even generate and test novel (‘own’) modulation strategies. During functional-localizer and no-feedback runs, participants were asked to try to evoke different brain-activation levels based on their current hypothesis on how the BOLD-signal magnitude can be altered systematically.

### Data acquisition

#### (f)MRI data acquisition

(f)MRI data were obtained using a 1.5-T whole-body (Magnetom Sonata; Siemens AG, Erlangen, Germany) and a 3-T head scanner (Siemens Allegra, Siemens AG) (see Table 1). Participants were placed comfortably in the MRI scanner and their heads were fixated with foam padding to minimize spontaneous or task-related motion.

##### Functional measurements

Repeated single-shot echo-planar imaging (EPI) was performed using the BOLD effect as an indirect marker of local neuronal activity (Ogawa et al., 1990). Except for the number of acquisitions (functional-localizer run: 273 volumes; modulation runs: 247 volumes), identical scanning parameters were used for all functional measurements (repetition time [TR] = 2000 s, echo time [TE] = 40 ms, flip angle [FA] = 90°, field of view [FOV] = 224 × 224 mm^2^, matrix size = 64 × 64, number of slices = 25, slice thickness = 3 mm, 1-mm gap, slice order = ascending/interleaved).

In the feedback sessions, functional images were reconstructed and written to the scanner console’s hard disk in real time using a custom-made image export running on the image reconstruction computer (implemented in Siemens ICE VA30) (Weiskopf et al., 2004a, Weiskopf et al., 2005). The real-time data analysis software (see below) running on a separate PC retrieved the image files via local area network (LAN) and a Windows drive map as soon as they were created by the image reconstruction system.

##### Anatomical measurements

Each participant underwent a high-resolution T1-weighted anatomical scan using a three-dimensional (3D) magnetization-prepared rapid-acquisition gradient-echo (MPRAGE) sequence (1.5-T scanning: 192 slices, slice thickness = 1 mm, no gap, TR = 2000 ms, TE = 3.93 ms, FA = 15°, FOV = 250 × 250mm^2^, matrix size = 256 × 256, total scan time = 8 min and 34 s; 3-T scanning: 192 slices, slice thickness = 1 mm, no gap, TR = 2250 ms, TE = 2.6 ms, FA = 9°, FOV = 256 × 256mm^2^, matrix size = 256 × 256, total scan time = 8 min and 26 s).

#### Acquisition of physiological data

In order to assess potential cardiorespiratory effects on the fMRI signal level, heart and breathing rates of the participants were recorded during feedback runs using the scanner’s standard MRI-compatible pulse oximeter and chest band. Due to technical limitations, acquisition of physiological data was only feasible during 1.5-T measurements (five participants).

#### Acquisition of introspective data

After MRI scanning, participants filled in a *post-hoc* questionnaire obtaining precise descriptions of the applied activation and modulation strategies as well as other relevant information (*e.g.*, subjective experience with neurofeedback).

### Data analysis

#### (f)MRI data analysis

##### Online/real-time analysis of fMRI data

Functional data of the feedback session were analyzed using real-time data analysis software (Turbo-BrainVoyager, Brain Innovation B.V., Maastricht, the Netherlands) in order to a) select and define the neurofeedback target region and b) generate the neurofeedback information.

*Selection and definition of neurofeedback target regions:* After completion of the functional-localizer run, the first two volumes were discarded from further analysis to account for T1-saturation effects. Functional data were then pre-processed (motion correction, linear-trend removal, temporal high-pass filtering [three cycles/time course]). Eventually, a multiple-regression general linear model (GLM) was calculated voxel-wise applying predictors corresponding to the two target-level conditions (predictor time courses being derived from a boxcar function convolved with a standard hemodynamic response function [single-gamma function (Boynton et al., 1996)].

Candidate neurofeedback target regions were identified by contrasting the mean brain activation during both target-level conditions to the mean activation during the interleaved resting periods. From the obtained F-maps (*p* < 0.05, Bonferroni-corrected), a region of interest (ROI) was defined for each participant individually as neurofeedback target region based on the following criteria:

(1) The region’s BOLD-signal time course should be reliable and robust, demonstrating a typical hemodynamic response shape across the entire functional run and small standard errors when averaging across repetitions.
(2) The region should present a strong fMRI response (high BOLD-signal change relative to baseline and high signal-to-noise ratio).
(3) Brain regions should be known to be involved in the performance of the selected activation strategy, *e.g.*, Broca’s area during inner speech (Shergill et al., 2002) or premotor areas during motor imagery (Guillot et al., 2008) should be preferred (implementation of *a priori* knowledge).
(4) The region should be relatively insensitive to susceptibility artifacts.
(5) The region should comprise about 10–15 neighboring voxels across up to three separate fMRI slices.

Maximal % BOLD-signal values of the selected neurofeedback target regions were calculated and noted down as they were needed for calculating the neurofeedback information.

*Generation of the neurofeedback information:* After the first two volumes were discarded from further analysis, the data of the feedback runs were analyzed in real time. The computational steps described in the following were performed as soon as the necessary data were available and had been spatially aligned to the first volume of the functional-localizer run to correct for potential head movement. In order to generate the neurofeedback information, a baseline was determined as the mean of the five data points prior to the onset of the first modulation trial. The baseline was continuously updated before each new modulation trial (sliding baseline). Eventually, the neurofeedback information was calculated separately for each functional volume by:

(1) Extracting and averaging the BOLD-signal values of all voxels composing the neurofeedback target region.
(2) Normalizing the resulting mean value to % BOLD-signal change with respect to the corresponding baseline level.
(3) Calculating the level ratio (LR) by relating the % BOLD-signal change value of the current time point *i* (% BOLD_i_) to the maximal % BOLD-signal value (% BOLD_max_) obtained from the functional-localizer run (LR = % BOLD_i_/% BOLD_max_). The resulting value was clipped to the range [0.0–1.0] corresponding to the baseline level and the maximum level achieved in the functional-localizer run, respectively. Values below 0.0 and above 1.0 were displayed as 0.0 and 1.0, respectively.
(4) Relating the level ratio to the number of rectangles to be colored gray (N_filled_) by linear transformation (N_filled_ = round (10 × LR)).

Thus, an activation level of half the maximum activation (LR = 0.5), for example, was represented by five gray rectangles (filled from bottom) within the thermometer-like neurofeedback display. Neurofeedback information was immediately presented to the participant and was continuously updated every 2000 ms (*i.e.*, every each functional volume).

##### Offline analysis of (f)MRI data

*Post-hoc* analysis of the (f)MRI data was done using BrainVoyager QX (v2.8, Brain Innovation, Maastricht, the Netherlands) and SPSS 21 (SPSS Inc., Chicago, IL, USA).

###### Analysis of anatomical data

Obtained anatomical data sets were first corrected for spatial intensity inhomogeneity. For each participant, the data set from the first session was transferred into ACPC space. Subsequently, the data set from the second session was automatically aligned to the ACPC version of the first data set. Finally, both data sets were spatially normalized by Talairach transformation.

###### Analysis of functional data

Pre-processing. All functional data sets underwent standard pre-processing optimized for the current experiment. Slice scan-time correction and temporal high-pass filtering (three cycles per time course) was performed to account for temporal differences in slice acquisition and to remove low-frequency drifts, respectively. Furthermore, 3D head-motion detection and correction was applied by spatially aligning all functional volumes of a session to the first functional volume of the first run within that session. Finally, all functional runs were spatially normalized to Talairach space and interpolated to a 3-mm^3^ voxel resolution. The individual neurofeedback target regions were transformed into 3D volumes of interest (VOIs) in Talairach space.

Group analysis of mean betas. A VOI-based random-effects group GLM analysis (standard feature implemented in BrainVoyager QX) was carried out. The GLM included predictors for each target-level condition (low, medium, and high), type-of-training condition (no feedback and feedback), and six motion parameters (three rotations and three translations) as confounding predictors. Condition effects were modeled using a boxcar function, which was convolved with the Two-Gamma hemodynamic impulse function (Friston et al., 1998) to take into account the hemodynamic response delay. Beta values for each target-level condition were calculated separately for each type-of-training condition by fitting the GLM to the average BOLD-signal time course within the individual VOIs. Based on the resulting betas, a two-way repeated measures analysis of variance (ANOVA, *F*-Test) with factors for target-level and type-of-training was performed to test the interaction hypothesis. Furthermore, a contrast analysis was carried out, specifically testing the differences between target-level conditions within a type-of-training condition. Obtained p-values were evaluated against a one-sided threshold of *α* = 0.05, as a directed hypothesis of the *gradual self-regulation* and *instantaneous feedback effect* was posed *a priori*.

Single-trial analysis. In order to extract single-trial beta values for all modulation (no-feedback and feedback) trials, a VOI-based GLM (including the six motion predictors as confounding predictors next to the single-trial predictors) was carried out separately for each functional run. Single-trial beta values were calculated by fitting the GLM to the average BOLD-signal time course within the individual VOIs. The resulting single-trial beta values were correlated (Pearson’s correlation coefficient) with the particular target level for both type-of-training conditions separately and a Fisher z-transformation was applied to each correlation coefficient. This resulted in two Fisher z-transformed correlation coefficients for each participant, one for the no-feedback and one for the feedback condition. Subsequently, a one-sided paired t-test was carried out, comparing the correlation coefficients between the two sessions against a threshold of *α* = 0.05 (reasoning see above).

Single-subject analysis. In order to test the ability of each individual participant to gradually modulate regional brain activation to the target levels in the no-feedback and feedback condition, the individual VOI-specific beta values for each combination of target-level and type-of-training conditions were entered in a contrast analysis, testing whether the brain-activation levels can be significantly differentiated in both the type-of-training conditions. Obtained p-values were evaluated against a one-sided threshold of *α* = 0.05.

#### Analysis of physiological data

Acquired heart and breathing rates were analyzed using in-house software written in MATLAB (v6.5 R13; The MathWorks, Natick, USA). Mean values and standard errors were calculated separately for the target-level conditions and the resting condition.

#### Analysis of introspective data

The *post-hoc* questionnaires that participants filled in after each MRI scanning session were qualitatively analyzed to gain insights in the participant’s phenomenological experience of the selected activation and modulation strategies.

## Results

### Introspective results

The individually chosen activation strategies (mental tasks; see Table 1) considerably varied across participants and can be classified into four categories: inner speech, motor imagery, auditory imagery, and visual imagery.

#### Inner speech

Five participants (P01, P03, P05, P07, P10) chose inner speech as their activation strategy. They either recited a given text (*e.g.*, a poem or prayer) or spontaneously generated speech silently. Strategies to modulate the BOLD-signal level included: a) making the content of the inner speech more complex (naming single words to generating whole sentences), b) speaking at a different pace (very slow to extremely fast), or c) varying sound intensity (almost silent to extremely loud).

*Motor imagery.* Two participants performed a motor-imagery task (mental drawing [P06] and mental running [P09]). Modulation strategies involved systematically varying the rhythm of the movement, the environment in which the movement was embedded (*e.g.*, from running in a calm environment to running together with several people, culminating in running in a competition), and the pace of movement.

#### Auditory imagery

Two participants performed auditory imagery. One participant (P02) mentally conducted an orchestra (mental orchestra) and changed the pace, rhythm and sound level of the music as well as number of orchestra instruments to vary the BOLD-signal level. The second participant (P08) imagined simple sounds (mental sounds) varying the rhythmicity of the tones (no rhythm to high rhythmical variations) in order to adapt the BOLD-signal level.

#### Visual imagery

One participant (P04) performed visual motion imagery*.* The participant imagined a vertically jumping object and changed frequency and rhythm of the object’s motion as modulation strategy.

In general, participants reported to have been able to apply their selected activation strategy easily and that it was possible to additionally apply modulation strategies (*i.e.*, vary the content of the imagination). They also indicated to have been able to attend and react to the neurofeedback-display changes in the feedback condition and that modifying the modulation strategy to some extent was also represented in a change in the neurofeedback signal. Generally, the feedback condition was perceived enjoyable though being more demanding and requiring more attentional resources. Especially the lowest modulation level seemed to be difficult to obtain for some participants. Most importantly, participants reported that some of the initial modulation strategies were quite effective but that the provision of neurofeedback helped them to further optimize (fine-tune) the modulation strategies or to even elaborate new strategies. For example, P03 using inner speech as activation strategy employed a systematic variation of the speech rate as modulation strategy. In the neurofeedback condition she realized that using an unnaturally low speech rate did not result in a low BOLD-signal level. Accordingly, she adapted her initial modulation strategy – using finally a normal, fast and very fast speech rate to achieve a low, medium and high BOLD-signal level, respectively.

### fMRI results

#### Neurofeedback target regions

**Fig. 3:**
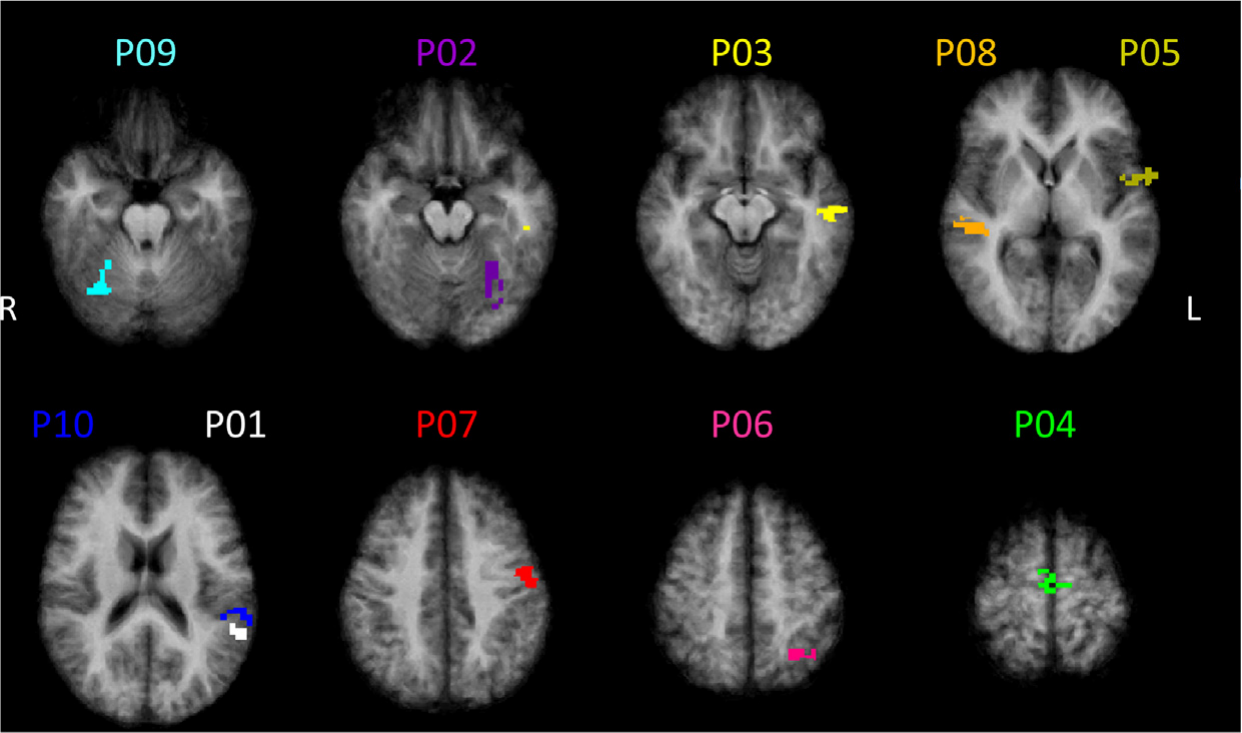
Individual neurofeedback target regions. The figure shows the individually defined neurofeedback target regions overlaid on transversal slices of the participants’ mean anatomy in Talairach space. Note that the selected regions are the widely distributed across the whole cortex. Characteristics of the selected brain regions (anatomical labeling, size, Talairach coordinates *etc.*) can be derived from Table 2. *Remarks:* L = left hemisphere; R = right hemisphere.

For each participant, a neurofeedback target region fulfilling the above-mentioned criteria could be determined based on the functional-localizer data obtained during the feedback session (see Fig. 3). Characteristics of the selected ROIs (anatomical labeling, size, Talairach coordinates *etc.*) can be derived from Table 2.

**Table 2:** Characteristics of neurofeedback target regions

Participant	Anatomical label	Size (mm^3^)	Talairach coordinates (*x*, *y*, *z*)			Maximal % BOLD-signal value
P01^∗^	STG	511	−57	−41	16	5.0
P02	FG	673	−27	−59	−15	2.0
P03	MTG	654	−53	−18	−8	2.0
P04	SFG	471	0	−11	−59	2.0
P05	IFG	481	−56	3	4	3.0
P06	SPL	614	−31	−55	50	4.0
P07	preCG	447	−49	−8	41	3.0
P08	STG	774	48	−26	4	2.0
P09	FG	526	28	−57	−19	5.0
P10	SMG	882	−51	−33	17	2.0

*Remark:*^∗^left-handed. *Abbreviations:* FG, fusiform gyrus; IFG, inferior frontal gyrus; STG, superior temporal gyrus; MTG, middle temporal gyrus; preCG, precentral gyrus; SFG, superior frontal gyrus; SMG, supramarginal gyrus; SPL, superior parietal lobule.

#### Gradual self-regulation effect (no-feedback data)

##### Group results

**Fig. 4:**
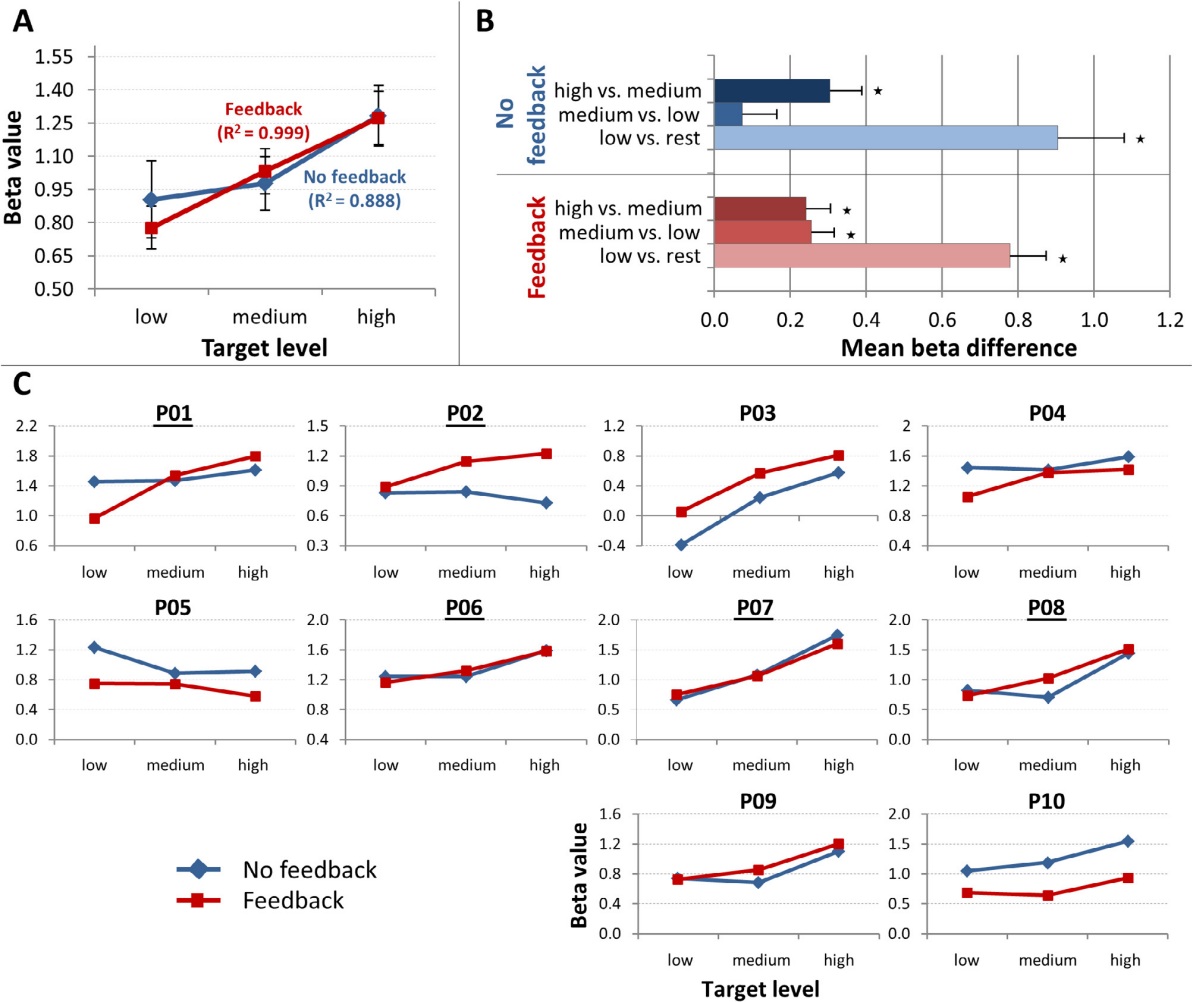
Gradual self-regulation ability across both type-of-training conditions (group and single-subject results). A. Mean beta values for each target-level condition across all participants separately for the no-feedback (blue) and feedback (red) condition. Error bars represent standard errors of the means from within-subjects analysis. B. Contrast analysis between target level-specific beta values separately for the no-feedback and feedback condition across all participants. All comparisons reach statistical significance (*p* < 0.01, see asterisks) except for one contrast (contrasting the medium *vs.* the low target level in the no-feedback condition). C. Single-subject mean beta values separately for each target-level and type-of-training condition. Participants with a black underline underwent feedback condition first and no-feedback condition second.

Across participants, mean beta values significantly increased with each target-level step in a linear way (*p* < 0.001; see Fig. 4A, B). Fitting a linear trendline to the obtained mean target-level beta values showed a clear linear modulation of the brain-activation level (*R*^2^ = 0.888, see Fig. 4A). Moreover, contrast analyses showed that two hypothesized between-target level contrasts were significant (high *vs.* medium level and low level *vs.* resting; *p* < 0.01). No difference could be obtained for contrasting the medium *vs.* the low target-level condition (*p* = 0.22, Fig. 4B).

##### Single-subjects results

When looking at the number of significant between-target level contrasts (in the desired direction), 80% of participants (P01, P03, P04, P06-10) were able to gradually modulate local brain activation to at least two target levels in the no-feedback condition (see second column of Table 3). Using a stricter criterion, namely a significant correlation of the single-trial beta values and the target levels, actually 60% of participants (P03, P06-P10) were able to gradually modulate the brain-activation level in the absence of neurofeedback (see third column in Table 3). In Fig. 4C, mean beta values of the no-feedback conditions are plotted separately for each participant.

**Table 3:** Number of significant between-target level contrasts and correlations of single-trial beta values and target levels separately for the two type-of-training conditions per participant

Participant	No-Feedback condition		Feedback condition	
	Number of significant contrasts	Correlation of single-trial beta values and target levels	Number of significant contrasts	Correlation of single-trial beta values and target levels
P01	2	0.188	3 (+)	0.745^∗∗^ (↑)
P02	1	−0.083	2 (+)	0.355^∗^ (↑)
P03	2	0.415^∗∗^	2 (=)	0.677^∗∗^ (↑)
P04	2	0.094	2 (=)	0.402^∗∗^ (↑)
P05	1	−0.326^(∗∗)^	1 (=)	-0.152 (↑)
P06	2	0.287^∗^	3 (+)	0.411^∗∗^ (↑)
P07	3	0.778^∗∗^	3 (=)	0.824^∗∗^ (↑)
P08	2	0.455^∗∗^	3 (+)	0.495^∗∗^ (↑)
P09	2	0.531^∗∗^	2 (=)	0.41^∗∗^ (↓)
P10	2	0.501^∗∗^	2 (=)	0.266 (↓)
Mean	1.9	0.284	2.3	0.443

Remarks:^*^p < 0.05 (desired direction); ^**^p < 0.001 (desired direction); ^(**)^*p* < 0.001 (undesirable direction); participants with a black underline underwent feedback condition first and *no-feedback condition* second; (↑) or (↓) indicates a higher or a lower correlation coefficient in the feedback condition (*vs.* in the no-feedback condition); (+) or (=) indicates more or the same number of significant between-*target level* contrasts.

#### Instantaneous feedback effect

##### Single-subjects results

**Fig. 5:**
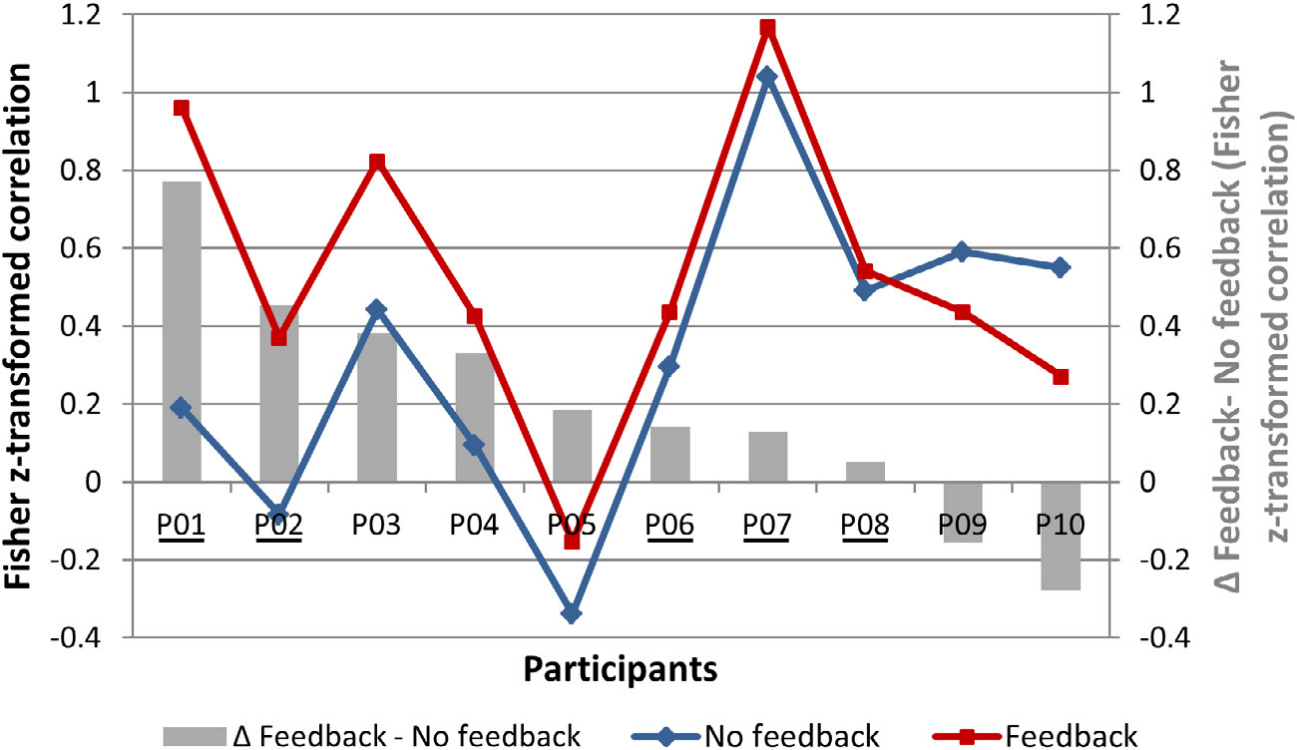
Comparison of individual gradual self-regulation ability across the two type-of-training conditions. The figure depicts individual Fisher z-transformed correlation values between obtained single-trial beta values and desired target levels separately for the no-feedback (blue line) and feedback (red line) condition and their differences (gray bars). In 80% of the participants, single-trial beta values were more correlated with the desired target levels when participants received neurofeedback (*vs.* being not provided with neurofeedback information). Participants with a black underline underwent feedback condition first and no-feedback condition second). (For interpretation of the references to colour in this figure legend, the reader is referred to the web version of this article.)

Four participants showed a higher number of significant between-target level contrasts in the feedback compared to the no-feedback condition (P01, P02, P06, P08). The remaining six participants (P03–P07, P09, P10) showed the same amount of significant between-target level contrasts for both type-of-training conditions (see fourth column in Table 3). Calculating the differences between the Fisher z-transformed correlations (of the single-trial beta values and the activation target levels) for the feedback and no-feedback data show that in 80% of the participants, receiving neurofeedback (*vs. not* receiving neurofeedback) led to an increased association between the single-trial modulation of regional brain activation and the desired target level (see Fig. 5 and fifth column of Table 3).

In Fig. 4C, mean beta values for both the no-feedback and the feedback conditions are plotted separately for each participant. One participant (P05) did not seem capable of performing target level-specific adjustments of the BOLD-signal level independent of type-of-training condition. However, neurofeedback seemed to weaken the negative association observed in the no-feedback condition (see Fig. 4C, P05). In two participants (P09 and P10), providing neurofeedback seemed to rather impede the ability to gradually modulate the brain-activation level which these participants were presenting in the (preceding) no-feedback condition (see Table 3, P09 and P10).

##### Group results

In the ROI-based random-effects group analysis, the interaction effect between the factors target level and type of training just missed significance (*p* = 0.083). However, the correlation of the single-trial beta values and the activation target levels was significantly higher in the feedback than in the no-feedback condition (*p* < 0.05). Fitting a linear trendline to the target-level beta values across participants showed that providing neurofeedback led to an almost linear modulation of brain activation within the neurofeedback target regions (*R*^2^ = 0.999), while in the no-feedback condition, the linear modulation was lower to some degree (*R*^2^ = 0.888) (see Fig. 4A). This becomes also obvious by inspecting Fig. 4B showing increased equidistance of target-level beta-value differences in the feedback (*vs.* no-feedback) condition. Finally, contrast analyses showed that in the feedback condition, each target-level beta value was significantly different from the other target-level beta values and from rest (*p* < 0.002, Fig. 4B) while in the no-feedback condition, only two of these contrasts were significant (see above).

#### Heart and breathing rates

**Fig. 6:**
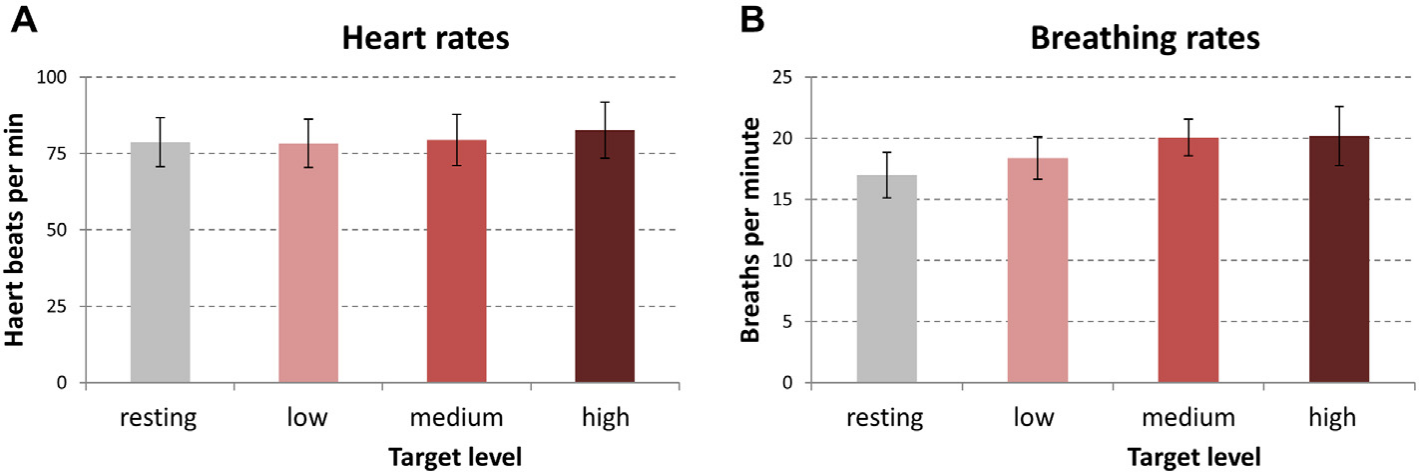
Mean heart and breathing rates for each target-level condition. Mean heart (**A**) and breathing (B) rates of P02-P05 and P09 are plotted separately for each target-level condition. While mean heart rates only showed negligible differences across target-level conditions, slightly increased breathing frequencies at higher target-level conditions can be observed. Error bars indicate variance across participants (±SEM).

In Fig. 6, mean heart and breathing rates obtained during the different feedback conditions are plotted jointly for P02–P05 and P09 (with all values being in the normal range). While observed differences in heart rate across target-level conditions were extremely weak, slightly augmented breathing frequencies were detected for higher target-level conditions on a descriptive level.

## Discussion

The current feasibility study systematically investigated the possibility to gradually modulate the BOLD-signal level within mental task-related brain regions as well as the potential benefit of providing neurofeedback information in this context. Ten healthy participants underwent two (f)MRI sessions in which they were asked to reach three different levels of brain activation by a) applying activation and modulation strategies alone or b) additionally considering neurofeedback about their current brain-activation level within a region of interest to optimize their gradual self-regulation performance. The obtained results demonstrate that participants can indeed gradually modulate their brain activation when using appropriate cognitive strategies. Moreover, additionally providing participants with continuous neurofeedback information can further enhance the gradual self-regulation performance.

### Self-regulation of regional brain activation to different target levels

#### Remarkable gradual self-regulation ability based on suited cognitive strategies

In the first part of our study, we investigated whether humans are generally (*i.e.*, without getting neurofeedback information) capable of modulating regional brain activation gradually (as opposed to an ‘all-or-none’ strategy employed in previous studies) based on given activation and modulation strategies that emerged from literature and/or intuition of the experimenters (*gradual self-regulation effect*).

Generally, when looking at the no-feedback results, participants succeeded in up-regulating their brain-activation level within the activation strategy-related brain region to a great extent (see Fig. 3A, C and maximal % BOLD-signal values in Table 2) and were able to modify their cognitive strategies in such a way that, on the group level, the reached brain-activation level significantly increased with each target-level step in a linear fashion (Fig. 4A) and two different target levels could be significantly distinguished (Fig. 4B). The respective single-subject results show that individual participants were well-skilled to gradually self-regulate their brain-activation level: 80% of participants were able to gradually modulate local brain activation to at least two different target levels and in 60%, even a significant correlation of the single-trial beta values and the activation target levels could be ascertained (Table 3, Fig. 4A–C).

The remarkable gradual self-regulation performance of our participants might be caused by the following reasons:

1. As established activation strategies were suggested to the participants, they all chose an immediately successful (cognitive) activation strategy (see Table 1) that generally evoked robust regional brain activation (maximal % BOLD-signal level of the functional-localizer data within the region of interest were ≥2.0 in all participants, Table 2). This made it more likely to efficiently apply modulation strategies and to reach intermediate brain-activation levels.
2. The information about the potential modulation strategies given to the participants before the MRI session was highly relevant for successfully performing the gradual self-regulation task – indeed resulting in the desired BOLD-signal variations when participants carefully followed these instructions.
3. As half of the participants underwent the feedback condition first and the no-feedback condition in a second step, the obtained *gradual self-regulation effect* might be partially explained by a *neurofeedback-transfer effect* (applying successful modulation strategies refined during the earlier feedback condition in the later no-feedback condition). However, this seems unlikely as the majority of the participants starting with the no-feedback condition demonstrated already a clear gradual modulation ability in the no-feedback situation.

Overall, our results are in accordance with previous findings showing that the BOLD-signal level can be modulated temporally by varying certain aspects of mental-task performance (*e.g.*, rate of inner-speech generation [Shergill et al., 2002], rate of imagined movements [Berman et al., 2012], increased angles of mental rotation [Lipp et al., 2012] or particular cognitive processes as object-based attention [*e.g.*, Culham and Kanwisher, 2001]).

The applied activation and modulation strategies as well as the selected regions of interest varied considerably across participants, with no clear advantage for any strategy or brain region (see Table 1, Table 2, Fig. 4C). This implies that gradual self-regulation of brain activation can, in principle, be achieved using various activation and modulation strategies and at (at least) several brain locations.

#### Enhanced gradual self-regulation ability through neurofeedback

In a second step of this study, we investigated whether additionally providing neurofeedback information on the current brain-activation level can further improve the gradual self-regulation performance (*instantaneous feedback effect*).

The remarkable gradual self-regulation ability already obtained in the no-feedback condition (most probably caused by optimal instruction of the participants; see discussion above) made it actually quite challenging to demonstrate an *instantaneous feedback effect* on top of this *gradual self-regulation effect*. Despite this, single-trial analysis showed that most participants demonstrated a higher gradual self-regulation performance in the feedback condition compared to the no-feedback condition (see increase of correlations of observed single-trial beta values and target levels in Fig. 5 and Table 3) suggesting a benefit of providing neurofeedback on a single-subject level. These single-subject results were jointly analyzed on the group level (paired t-test) showing a significant *instantaneous feedback effect* meaning that providing neurofeedback can indeed further enhance the ability to gradually modulate regional brain activation. Moreover, as can be obtained from Table 3, more significant between-target level contrasts (in the desired direction) could be differentiated in the feedback condition (*vs.* in the no-feedback condition) in four participants (Table 3) and on the group level (see three significant between target-level contrasts in the feedback *vs.* only two significant contrasts in the no-feedback condition displayed in Fig. 4B). Finally, using a stricter and more sensitive criterion, namely a significant correlation of the single-trial beta values and the activation target level, actually 80% of the participants were able to gradually modulate the brain-activation level in the feedback condition (*vs.* 60% of participants in the no-feedback condition; see Table 3). All these results indicate that the additional availability of neurofeedback about the current BOLD-signal level can indeed facilitate the gradual self-regulation performance and therewith confirms our second hypothesis.

The observed *instantaneous feedback effect* cannot be explained by a trivial *training effect* (increased gradual self-regulation performance simply caused by repeated mental-task performance *vs.* successful use of neurofeedback information (*cf.* Sulzer et al., 2013b), as we employed an experimental design that included balancing the type-of-training conditions across participants. Additionally, the fact that the two participants who showed a lower correlation coefficient in the feedback (*vs.* the no-feedback) condition first underwent the no-feedback condition, speaks against a simple *training effect*. Another argument that suggests a real (*instantaneous*) *feedback effect* is that all participants starting with the feedback condition performed worse in the following no-feedback condition. Note, that the latter clearly points to an *instantaneous feedback* rather than a *feedback-transfer* effect, *i.e.*, the increased gradual self-regulation ability seems to be bound by the feedback situation and cannot be easily transferred to the no-feedback situation.

Though being significant on a group level, the *instantaneous feedback effect* is admittedly relatively small. Note, however, that if we would not have provided our participants with explicit activation and modulation strategies, this effect would have been most likely larger. We intentionally followed the described procedure (optimal cognitive preparation of the participants) as we aimed at investigating the specific effect that can be attributed solely to the presence of neurofeedback. Note also, that the *instantaneous feedback effect* had to be established on top of the considerable *gradual self-regulation effect* (see discussion above). Thus, it was not trivial to demonstrate an *instantaneous feedback effect*, especially when considering that individuals might not immediately benefit from neurofeedback. Processing neurofeedback information (in parallel to mental-task execution) strongly increases workload (see results on introspective reports of participants). Thus, gradual self-regulation of the BOLD-signal level might be hampered by the demanding multi-tasking requirements associated with the processing of the neurofeedback (monitoring, interpreting, and accordingly implementing the feedback). This might have been the case for the two participants that were not able to use the neurofeedback information in order to improve their gradual self-regulation performance (P09 and P10; see Fig. 4C, Fig. 5). These participants (both starting with the no-feedback condition) probably needed substantially more time to get used to the more demanding (dual-task) feedback situation resulting in a performance drop in the first instance (see Table 3, Figs. 4C, 5). Note that the amount of neurofeedback training was rather limited in our study (one feedback session including only ∼30 min of neurofeedback training). Participants with initial difficulties, might benefit from extended neurofeedback training across multiple sessions (Frank et al., 2012, Linden et al., 2012, Canterberry et al., 2013, Haller et al., 2013, Hartwell et al., 2013, Li et al., 2013, Ruiz et al., 2013b, Auer et al., 2015, Cordes et al., 2015, Sherwood et al., 2016) or from employing an alternative (individually tailored) neurofeedback display. Of course, it might be that some individuals will never benefit from neurofeedback or that providing feedback might even work disadvantageously for them. Note, that even if not all individuals benefit from rtfMRI neurofeedback, it might still constitute a crucial advancement on the individual level. All in all, we conclude that rtfMRI neurofeedback *can* enhance the gradual self-regulation ability.

### Reflections on task instruction in rtfMRI-neurofeedback studies

One current debate within the rtfMRI community concerns the way of instructing participants in neurofeedback experiments. In order to assure a true *feedback effect*, the practical realization of the feedback and the control condition(s) should solely differ in the presence of the (valid) neurofeedback information – implying that no other crucial difference between conditions should exist. Thus, a *feedback effect* should not be attributable to, *e.g.*, differences in task instruction but only to the presence of the neurofeedback information (Sulzer et al., 2013a, Thibault et al., 2016).

Note however, that in case a particular cognitive strategy has been shown to effectively alter brain activation in a wanted direction and this is paralleled with the desired behavioral change, it might be advisable to communicate this cognitive strategy to the participants before the rtfMRI-neurofeedback session in order to maximize positive effects. However, it might be then questionable whether a positive effect would actually be caused by the neurofeedback training or rather by the successful application of the specific instruction. In a study failing to replicate the pioneering work of deCharms et al. (2005) on positive rtfMRI-neurofeedback effects in chronic pain, participants were provided with identical task instructions (previously successful mental strategies in the same context) in both the feedback and the control condition and the same pain relief was observed in both conditions (unpublished data, discussed in Sulzer et al., 2013a). This suggests that the application of a suitable and to the participants’ well-communicated mental strategy can result in the same effects in the feedback and the control condition – speaking rather for an *effect of the mental strategy* and against a true *feedback effect* (Thibault et al., 2016).

In our current study, participants also received *identical* instructions concerning potential activation and modulation strategies in both the no-feedback and the feedback condition – therewith perfectly matching the two type-of-training conditions. As these strategies were generally very effective with respect to the purpose of the study (gradual self-regulation), we ascertained a remarkable *gradual self-regulation effect* already in the absence of neurofeedback. However and most importantly, we were able to demonstrate that participants’ performance was increased in the feedback condition which can only be attributed to the additional presence of the neurofeedback information, justifying the interpretation of a true (*instantaneous*) *feedback effect*.

Thus, in contrast to the replication study mentioned above we obtained differences between the no-feedback and feedback condition – indicating a true (*instantaneous*) *feedback effect* next to the *gradual self-regulation effect*.

### Potential for neuroscientific research and clinical applications

#### Potential applications for neuroscientific research

Classical fMRI studies employ the BOLD-signal level as the *dependent* variable in order to investigate cognitive, sensory, emotional or motor functions of the brain. In contrast, rtfMRI-based neurofeedback allows the use of the brain-activation level as the *independent* variable, allowing for an advanced investigation of brain functions. For example, the specific functional involvement of a particular brain region can be explored by self-regulating its activation and observing accordant behavioral changes (Weiskopf et al., 2003). The current study suggests that it might be possible to implement parametric designs for these purposes, which would constitute a powerful extension. Thus, further rtfMRI-neurofeedback research could investigate whether parametrically varied brain-activation levels are associated with accordant systematic perceptual, cognitive, emotional or behavioral changes.

#### Potential for clinical applications

##### BCI-based communication and control

For brain-based communication and control it is highly desirable to encode a particular intention on the single-trial basis. Previously, we have shown that this is feasible in an rtfMRI setup using information-encoding paradigms combining spatial and temporal BOLD-signal features (Sorger et al., 2009, Sorger et al., 2012). In the current study, we investigated the potential of using magnitudinal BOLD-signal features (*i.e.*, different brain-activation levels) for information encoding. When providing participants with appropriate activation and modulation strategies, we obtained medium to high correlations between the desired and the actually achieved brain-activation level for the majority of participants already in the no-feedback condition. Thus, even without implementing neurofeedback, employing magnitudinal BOLD-signal features might be feasible to neurally encode few information units (like “yes”/”no” or “up”/”down”). Note that this outcome is generally favorable in the BCI context as it indicates that the suggested novel information-encoding approach might qualify for BCI applications not requiring neurofeedback implementations which are technically much more challenging. Still, our second outcome, namely that the gradual self-regulation ability can be further enhanced by additionally providing neurofeedback information is absolutely desired as the observed gradual self-regulation performance was far from being perfect – especially when looking at the single-trial level. Note however, that averaging fMRI activation across multiple trials constitutes a powerful option to increase the BOLD-signal’s robustness, which has been successfully applied in healthy participants (averaging across three trials; Yoo et al., 2004, Lee et al., 2009) and in patients (averaging across five trials; Monti et al., 2010, Naci et al., 2012). Trial averaging, of course, results in a considerably lower information transfer rate (*a fortiori* taking into account the relatively long information-encoding time in fMRI-based BCIs owed to the sluggishness of the hemodynamic brain response). Note however, that the averaging approach might still constitute a valuable option for patients that do not have any other communication and control means left.

Future research might focus on intensive single-case studies (as, for example, performed in Weiskopf et al., 2003) systematically investigating the number of employable BOLD-signal levels starting with only two (extreme) target-level conditions and only introducing more target levels when two levels can be sufficiently differentiated (adaptive procedure). In this context, it might be beneficial to start with a neurofeedback training aiming at maximizing the BOLD-signal magnitude in the region of interest as initially reaching higher brain-activation levels would most probably increase the ability to (learn to) self-regulate intermediate brain-activation levels reliably (increased activation range).

All in all, we think that the suggested approach is promising even if the gradual self-regulation ability might be limited to a few levels. Note, that a differentiation of two BOLD-signal levels on a single-trial basis would already provide a considerable increase in degrees of freedom in hemodynamic BCI applications, namely when combined with the other employed approaches (*e.g.*, implementing additionally *spatial* and/or *temporal* BOLD-signal features for information encoding).

##### Neurofeedback therapy

The demonstration of an increased gradual self-regulation ability by means of neurofeedback not only advances BCI research, but might also extend the current spectrum of neurofeedback-therapy paradigms. So far, neurofeedback studies on clinical populations have focused on a maximal up- or down-regulation of regional brain activation as this has been thought to result in a maximal benefit (*i.e.*, a maximal reduction of clinical symptoms).

Note however, that employing a *parametric* modulation approach in this context might facilitate developing a general understanding of how regional brain activation can be influenced. More particularly, self-regulating brain activation to specific target levels might help fine-tuning the applied cognitive strategies in a faster fashion – leading to a steeper learning curve. Moreover, the possibility to reach different activation levels and to gain a more detailed sense for controlling activation in a targeted brain region might enhance the subjective feeling of success and the experience of self-efficacy – being of high importance from a motivational point of view.

Taking all these points together, we think that the parametric modulation approach as introduced in the current study might be a significant asset in the context of neurofeedback therapy. However, this possibility has to be systematically investigated in future studies.

### Potential confounding factors and limitations of the study

The results of the current study might be confounded by several factors that will be discussed below together with other limitations of the study.

#### Specific study population

Study participants were all students or staff members of the *Faculty of Psychology and Neuroscience* at *Maastricht University.* Thus, most of them had experience in participating in fMRI experiments. Moreover, they all had specific background knowledge on neuroscience (*e.g.*, with respect to neuroimaging, neurofeedback, BCI methodology *etc.*). However, we do not consider this knowledge to account for the ascertained *instantaneous feedback effect* as neurofeedback learning refers to the *practical* experience only gained in a neurofeedback situation itself. Moreover, participants could have applied potential pre-knowledge in both experimental conditions (feedback and no-feedback condition). Thus, we do not think that this pre-knowledge had an effect on the feedback condition only (and thus constituted a confounding factor that could account for the *instantaneous feedback effect* established in this study). Note however, that relevant methodological pre-knowledge (*e.g.*, in our case, on how to systematically elaborate the best modulation strategy) might constitute a facilitating factor for the neurofeedback-learning *process* and it might be advisable to always provide participants if possible with potentially helpful information. But as this refers to knowledge that cannot be acquired practically in a (preceding) neurofeedback session, it cannot account for the ascertained *instantaneous feedback effect*.

While our participants had the abovementioned pre-knowledge, they had not participated in a neurofeedback training (with either EEG, fMRI or fNIRS) before. Thus, they had no (in this context critical) *practical* neurofeedback experience that they could fall back on and could have transferred to the current neurofeedback session.

#### Limited number of participants

The number of participants in the current study is rather low (*n* = 10). One problem of small sample-sized studies is that the statistical power is low and that the sensitivity to outliers is higher than in studies with large sample sizes. Therefore, the generalization of obtained findings to the population has to be done with care. Note however, that a significant result obtained in a small sample-sized study (when well-controlled for false positives) is even more compelling evidence than the equivalent result with a larger sample-sized study (Friston, 2012). Thus, our results generally support that the gradual modulation approach is feasible in BCI and neurofeedback contexts and worth to be investigated in more detail and with larger sample sizes.

#### Not blinding participants

As discussed above, we considered it crucial to keep the information given to the participants before entering the scanner constant across all participants – independently of whether they started with the no-feedback or the feedback condition. Therefore, participants were not blinded and we relied on the assumption that our participants tried following the experimenters’ instructions to the best of their knowledge and belief.

#### Choice of control conditions for investigating the instantaneous feedback effect

Several control conditions to investigate an rtfMRI-neurofeedback effect have been suggested and implemented in the past, *e.g.*, providing no feedback (Auer et al., 2015), sham/pseudo feedback (deCharms et al., 2004, deCharms et al., 2005, Rota et al., 2009, Caria et al., 2010, Scharnowski et al., 2012), feedback from another brain region (deCharms et al., 2005) *etc.* We chose the no-feedback condition for the following reasons: Firstly, this condition was already implemented in our study design as, as a first step, we investigated the *principal* ability to gradually modulate brain activation (*i.e.*, without providing neurofeedback). Secondly, we considered the no-feedback condition as the most valid or appropriate control condition in the current BCI context as for BCI applications, providing sham/pseudo feedback would not constitute a meaningful option. Note, however, that BCI applications *without* involving a neurofeedback component might still constitute a reasonable alternative (see discussion above).

#### Limited amount of physiological data

Due to technical problems, heart and breathing data were only available for five out of ten participants and merely for the feedback condition (thus for in total 25% of the fMRI data). Because of the limited amount of physiological-data acquisitions, we were not able to systematically examine the effect of heart and breathing rates on the fMRI signal by adding physiological data as parameter of no interest. This would have constituted a valuable addition.

#### Cardiorespiratory effects

Analyzing available heart and breathing data revealed slightly increased breathing frequencies with higher target-level conditions (see Fig. 6B). Physiologically, an increase in the breathing frequency leads to a decrease of the carbon-dioxide (CO_2_) concentration and an increase of the oxygen (O_2_) concentration in the blood. Animal experiments systematically varying O_2_ concentrations demonstrated that hyperoxia (enhanced levels of O_2_ in the blood) actually leads to a decrease of BOLD-signal levels (Sicard and Duong, 2005, Wibral et al., 2007). In accordance with this, BOLD-signal increases have been reported in hypercapnia (enhanced levels of CO_2_ in the blood) (Kastrup et al., 1999). Both findings indicate that increased breathing frequencies should go along with *decreased* BOLD-signal levels (thus should rather work against our gradual self-regulation hypothesis).

To summarize, the latter theoretical consideration and our descriptive physiological results imply that the obtained differences in the obtained brain-activation magnitude across target-level conditions are unlikely to be driven by cardiorespiratory effects.

#### General arousal effects

Possible changes in general arousal are also unlikely to account for the obtained gradual self-regulation results. Explorative analysis of the fMRI data (results not shown) revealed no widespread activation increases for higher target-level conditions that would be expected in that case.

#### Study design

We implemented a within-subject design. Actually, a between-subject design would have had certain advantages, especially in terms of avoiding potential *feedback-transfer effects* (*e.g.*, through applying modulation strategies elaborated during earlier feedback runs in subsequent no-feedback runs in half of our participants). However, a between-subject design requires a considerably higher number of participants per group than could be realized within the scope of the current study. As the number of participants to be trained in rtfMRI-neurofeedback studies is limited in general, a within-subject design was considered more appropriate.

In order to address the discussed limitations and caveats of this study, several follow-up studies are requested. These more extensive studies should involve a considerably higher number of (naïve) participants, implement more fMRI sessions and advanced experimental designs (*e.g.*, alternating between no-feedback and feedback runs, involving more trials per participant, testing several visual feedback displays *etc.*) and include physiological parameters in the data analysis.

## Conclusions

The current study shows that humans – when being provided with appropriate activation and modulation strategies – are able to modulate the level of regional brain activation as measured with fMRI gradually. Moreover, we demonstrate that providing participants additionally with neurofeedback on the current BOLD-signal level within the target region can enhance the gradual self-regulation ability. Our findings were observed across a wide variety of activation strategies (mental tasks) and across clinical MR field strengths, indicating that these findings are robust and can be generalized across mental tasks and scanner types. Our study strongly motivates a further exploration of the novel parametric modulation approach that considerably enriches the current spectrum of rtfMRI-neurofeedback and BCI methodology which has attracted significant interest in fundamental and clinical neuroscience in the recent past.
